# Unveiling prognostic indicators in canine leishmaniosis: two decades of evidence

**DOI:** 10.1186/s13071-025-07042-0

**Published:** 2025-11-18

**Authors:** Juliana Sarquis, Carolina R. Sanz, Letícia Martins Raposo, Ana Montoya, Rocío Checa, Juan Pedro Barrera, Clara Gómez-Velasco, Efrén Estevez Sánchez, Guadalupe Miró

**Affiliations:** 1 Animal Health Department, Faculty of Veterinary Medicine, Complutense University of Madrid, Madrid, Spain; 2 Department of Quantitative Methods, Universidade Federal do Estado do Rio de Janeiro, Rio de Janeiro, Brazil

**Keywords:** Dog, *Leishmania*, Relapses, Outcome, Treatment response, Meglumine antimoniate, Miltefosine, Allopurinol, Logistic regression

## Abstract

**Background:**

Canine leishmaniosis (CanL), caused by *Leishmania infantum*, can be subclinical or present as a systemic, chronic, and potentially fatal disease. Treatment response in sick dogs is influenced by many factors associated with the host, the vector, and the environment. This study aimed to identify risk factors for poor prognosis in CanL, focusing on demographic, epidemiological, and clinical/clinocopathological variables.

**Methods:**

A retrospective analysis was conducted on a cohort of 300 dogs with CanL diagnosed between 2000 and 2022. Two logistic regression models were built to identify variables associated with an increased risk of relapses requiring repeated leishmanicidal treatments or of mortality due to CanL. A database with several variables was used to perform the study. These included demographic (age, sex, breed, and body weight), epidemiological (living conditions, travel history, and preventative measures), and clinical variables (clinical signs reported by the pet owner and physical examination findings, antibody titers, and LeishVet clinical stage), treatment history (first-line treatment, drug combinations), and outcome, among others. All analyses were conducted using R software and applying a significance level of 5% (*P* < 0.05).

**Results:**

Young dogs and those displaying weakness and ocular signs were more likely to develop relapses and require multiple leishmanicidal treatments, while weight loss was associated with a decreased risk. Additionally, dogs treated with miltefosine in combination with allopurinol as first-line treatment had a fivefold higher risk of needing multiple leishmanicidal treatments than those receiving meglumine antimoniate and allopurinol. Medium- to large-sized dogs had a fourfold higher mortality risk than small dogs, while this risk was almost 25 times higher in dogs in LeishVet stage IV compared with those in stage I. Mortality risk was also significantly higher in dogs displaying weakness, gastrointestinal signs, and lymphadenomegaly. Conversely, treatment with domperidone was associated with an 88% reduction in mortality risk.

**Conclusions:**

Our study highlights important risk factors for poor prognosis in CanL that should be carefully considered by clinicians and researchers when managing sick dogs, particularly regarding therapy decision-making.

**Graphical Abstract:**

**Figure:**
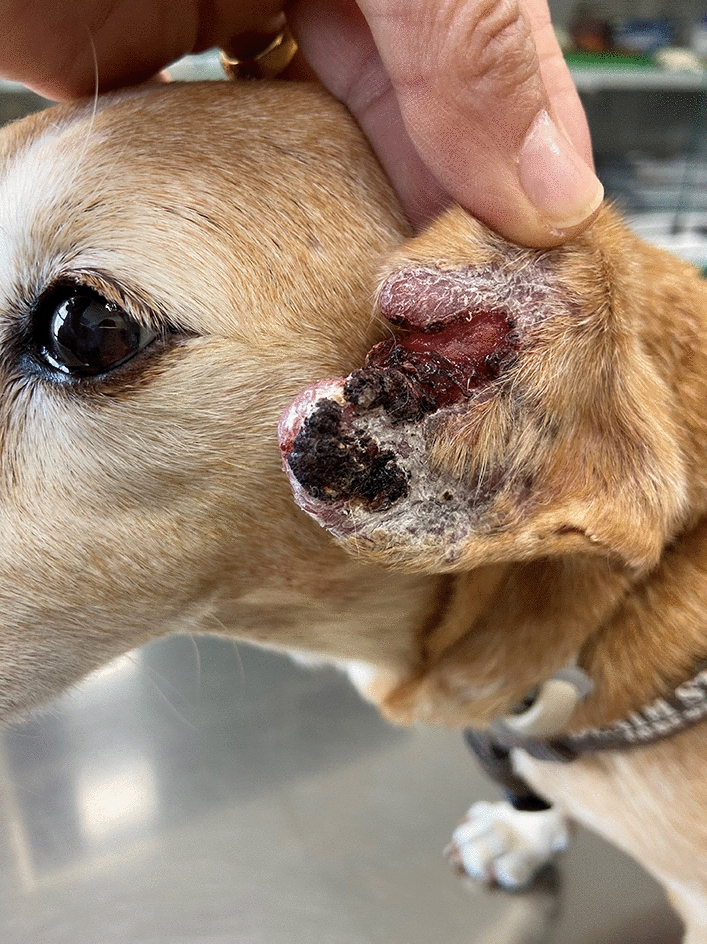

**Supplementary Information:**

The online version contains supplementary material available at 10.1186/s13071-025-07042-0.

## Background

Canine leishmaniosis (CanL), caused by *Leishmania infantum* and transmitted by phlebotomine sand flies, remains one of the most important vector-borne diseases affecting dogs globally. Following a bite from an infected sandfly, dogs may remain infected but clinically healthy for extended periods or develop a chronic and potentially fatal disease.

In Europe, the standard treatment for CanL involves the combination of meglumine antimoniate (MGA) or miltefosine (MIL) plus allopurinol (ALLO) [1, 2]. Current guidelines also recommend the use of immunomodulatory drugs, such as domperidone [3–5], as monotherapy in dogs with stage I disease, and dietary nucleotides (Impromune^®^) [6–9] as a substitute for ALLO in dogs with xanthinuria [10].

Treatment response to these drugs varies considerably [11, 12]. While some dogs experience long-lasting improvement in clinical signs and laboratory abnormalities, others show only partial or temporary improvement. Relapses, defined as the recurrence of clinical signs and/or clinicopathological abnormalities after an initial period of clinical improvement [13–15], are common in affected dogs, which often need repeated treatment cycles [16, 17]. However, distinguishing true clinical relapses that require a leishmanicidal treatment can be challenging, particularly in chronically sick dogs. The fact that dogs rarely achieve parasitological cure after treatment further complicates the assessment of treatment response in these animals.

Currently, no single diagnostic test is sensitive and specific enough to diagnose a clinical relapse in previously treated dogs [13]. For instance, antibody titers measured by immunofluorescent antibody test (IFAT) or enzyme-linked immunoassay (ELISA) may remain chronically elevated in dogs that do not present a clinical relapse or remain unchanged in those that present a relapse [17–19]. Regarding polymerase chain reaction (PCR), although quantitative PCR may provide an estimate of parasite burden and has shown promise in monitoring the disease [9, 20–22] and predicting relapses [21, 23], no standardized thresholds have been established to define clinically significant increases in parasitemia. Therefore, the assessment of treatment response should rely on combined criteria such as reduction in antibody titers, improvement in clinical signs and laboratory abnormalities after therapy, and the duration of disease-free intervals [12, 16, 24, 25]. Additionally, parasite detection by PCR can be useful in cases of uncertain serological results or in dogs that have not seroconverted [19, 21].

In CanL, poor treatment response should be considered if clinical signs and laboratory abnormalities persist for 3 months or more after initiating specific treatment [12], or if a relapse occurs requiring another course of leishmanicidal treatment in less than 12 months.

Previous research has identified multiple factors linked to poor treatment response and the occurrence of relapses in cutaneous and visceral human leishmaniosis, as detailed in a recent systematic review [26]. These factors include parasite characteristics (such as fitness, infectivity, and resistance mechanisms), vector-related aspects (including saliva components), host factors (immunological status, age, comorbidities, delayed diagnosis and treatment initiation, and compliance), and environmental factors.

Resistance to current therapeutic agents remains a significant cause of treatment failure in CanL [27–29]. Factors such as breed, genetic predisposition, and parasite virulence further influence therapeutic success or failure [30, 31]. Despite this, there are limited data on treatment outcomes and risk factors for unresponsiveness in CanL compared with human leishmaniosis.

Mathematical modeling has emerged as a powerful tool for predicting disease outcomes and guiding clinical decision-making in various medical fields, including infectious diseases [32]. In this study, we aimed to identify predictors of relapses and mortality in CanL using logistic regression modeling, focusing on demographic, epidemiological, clinicopathological, and therapeutic variables.

## Methods

### Selection of cases

Data were extracted from a database comprising records of 1141 dogs diagnosed with leishmaniosis between 2000 and 2022 at the Clinical Unit of Infectious Diseases of the Veterinary Teaching Hospital, Universidad Complutense de Madrid [31]. The diagnosis and clinical stage of CanL were established on the basis of a positive serology result (˃1:100) for *L. infantum* by IFAT, plus a positive cytology or a PCR result obtained from cutaneous lesions, bone marrow, or lymph node aspirates, together with the detection of clinical signs and/or clinicopathological abnormalities compatible with CanL, according to LeishVet guidelines [13].

Our database included detailed information on several variables [31], encompassing demographic and epidemiological data (e.g., age, sex, breed, body weight, living conditions, travel history, and use of preventative measures for CanL), clinical signs reported by the pet owners, physical examination findings, concurrent diseases, antibody titers measured by IFAT, LeishVet clinical stage, and treatment history (first treatment administered after diagnosis and drugs and treatment combinations used throughout the dogs’ lives), and follow-up duration.

### Data cleaning and preprocessing

Given the chronic presentation of CanL, a long follow-up period is necessary to evaluate outcomes. Therefore, the study included only cases with at least 2 years of follow-up or those that resulted in death or euthanasia due to CanL. Dogs that died of uncertain causes or from other diseases were excluded from the study.

Relapses were defined as the recurrence of clinical signs (e.g., cutaneous lesions, lymphadenomegaly, lethargy, and mucous membrane pallor) and/or clinicopathological abnormalities (e.g., anemia, hyperproteinemia, hyperglobulinemia, hypoalbuminemia, decreased albumin/globulin ratio, proteinuria, and azotemia), and a marked increase in antibody levels (more than twofold between monitoring samples), following an initial period of clinical improvement and requiring a new course of leishmanicidal treatment.

The outcome was based on the number of relapses, quantified by the number of leishmanicidal treatment cycles received per year (MGA plus ALLO or MIL plus ALLO), calculated by the sum of cycles received divided by the years of follow-up. Dogs that received < 1 cycle/year were classified as having a good outcome, while those that received ≥ 1 cycle/year were classified as having a poor outcome. For the survival analysis, dogs were divided into two groups: survived or died (death or euthanasia).

To retain sufficient power for the analysis, some variables were categorized: age (< 2 years: puppy, 2–4 years: young adult, 5–8 years: mature adult, ≥ 9 years: senior) [33], size (small: < 10 kg, medium: 10–29 kg, large: > 30 kg), and IFAT titer (< 1/200: negative, 1/200–1/400: low positive, 1/800–1/1600: medium positive, > 1/1600: high positive) [1, 13, 34, 35].

### Model building

**Fig. 1:**
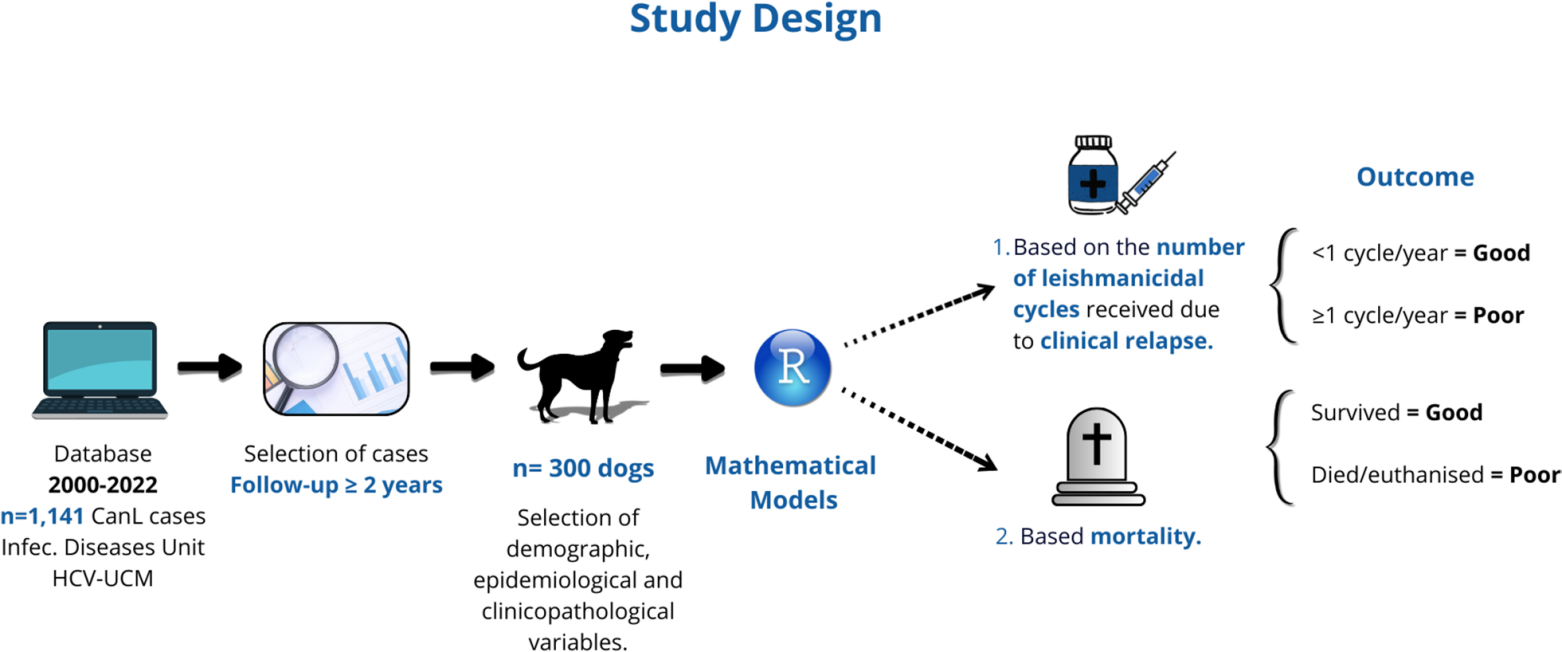
Study design and outcome classification

Two models were created to identify predictors of outcomes among the variables analyzed (Fig. 1). For model 1, the outcome variable measured the number of treatment cycles per year due to a clinical relapse, categorizing subjects into < 1 cycle per year and ≥ 1 cycle per year and excluding those labeled as died/euthanized. For model 2, the outcome variable categorized subjects into survived or died/euthanized.

In both models, variables that remained constant, those with dominant categories (frequency exceeding 95%), and variables related to the treatment scheme were removed. This step was crucial to avoid bias and ensure variability within the data. The presence of variables with near-zero variance can lead to unstable coefficient estimates, negatively affecting the interpretability and generalizability of models [36].

For both models, we used univariate logistic regression to identify variables associated with the outcomes, employing an initial *P*-value threshold of ≤ 0.25, as recommended by Bursac et al. [37]. These variables (Supplementary Tables 1 and 2) were then included in a multivariate logistic regression model. A stepwise regression approach was employed to enhance predictive accuracy and parsimony [38]. Specifically, backward elimination was used, where predictors were sequentially removed on the basis of their contribution to the model as measured by the Akaike information criterion (AIC). This iterative process continued until no further improvement in model fit could be achieved, as indicated by the AIC. The final models retained only those variables that significantly contributed to the prediction of the outcome.

To ensure the robustness of both models, multicollinearity was assessed using the variance inflation factor (VIF), with all VIF values being less than 2. Additionally, the goodness of fit of the final models was verified using the Hosmer–Lemeshow test [39]. The Hosmer–Lemeshow test assesses whether the observed event rates match the expected event rates in subgroups of the model population. A nonsignificant *P*-value indicates no significant difference between observed and predicted event rates, suggesting that the model fits the data well. This criterion was fulfilled in both models developed in this study. Statistical analyses were conducted using the R programming language through RStudio [40]. The significance level was set at *P* < 0.05.

## Results

### Study population

The study population comprised 300 clinical cases of CanL diagnosed between 2000 and 2022. Of these, 114 dogs (38%) were diagnosed between 2000 and 2010, and 186 (62%) between 2011 and 2022. Detailed demographic characteristics of the study’s population are displayed in Table 1.

**Table 1:** Age, sex, breed, and body size distribution of dogs (*n* = 300) diagnosed with CanL in our veterinary teaching hospital between 2000 and 2022

Characteristic	*n* = 300
Years	*n* (%)
2000–2010	114 (38.0)
2011–2022	186 (62.0)
Age group	
Puppy (< 2 years)	16 (5.3)
Young adult (2–4 years)	109 (36.3)
Mature adult (5–8 years)	127 (42.3)
Senior (≥ 9 years)	48 (16.0)
Sex	
Female	118 (39.3)
Male	182 (60.7)
Breed	
Crossbred	80 (26.7)
Purebred	220 (73.3)
Size	
Small (< 10 kg)	43 (14.4)
Medium (10–29 kg)	142 (47.5)
Large (> 30 kg)	114 (38.1)
Unknown	1

Age groups defined according to Creevy et al. [19]

Interestingly, only 3.7% of dogs (11/296) were vaccinated against leishmaniosis. Regarding other preventive measures, collars with proven repellence against sandflies were the most used (136/290, 46.9%), followed by spot-on treatments (115/291, 39.5%). Sprays were also applied in a few cases (13/293, 4.4%). Most dogs (191/289, 66.1%) were also treated against internal parasites with a broad-spectrum antiparasitic. Table 2 displays the epidemiological characteristics of dogs included in the study.

**Table 2:** Epidemiological characteristics of dogs included in the study

Characteristic	*n* = 300
Habitat	*n *(%)
Indoors	192 (65.8)
Outdoors	100 (34.2)
Unknown	8
Living with other pets	
Yes	20 (8.9)
No	205 (91.1)
Unknown	75
Vaccinated against leishmaniosis	
Yes	11 (3.7)
No	285 (96.3)
Unknown	4
Spot-on	
Yes	115 (39.5)
No	176 (60.5)
Unknown	9
Collar	
Yes	136 (46.9)
No	154 (53.1)
Unknown	10
Spray	
Yes	13 (4.4)
No	280 (95.6)
Unknown	7
Deworming	
Yes	191 (66.1)
No	98 (33.9)
Unknown	11

### Clinical and clinicopathological findings

Most dogs displayed weight loss (186/300, 62.2%), lymphadenomegaly (177/300, 60%), or cutaneous lesions (178/300, 59.5%). Additionally, more than half of the dogs presented physical weakness (171/300, 57.2%). Other common clinical signs included urinary (polyuria, polydipsia, urinary incontinence) (81/300, 27.3%), gastrointestinal (80/300, 26.8%), musculoskeletal (81/300, 27.1%), and ocular signs (61/300, 20.5%). Clinical signs and comorbidities displayed by the dogs included in the study are summarized in Table 3.

**Table 3:** Clinical signs and comorbidities observed in dogs with CanL included in the study (*n *= 300)

Clinical signs	*n* (%)	Comorbidities	*n* (%)
Weight loss	186 (62.2%)	Arthrosis	58 (19%)
Lymphadenomegaly	177 (60%)	Lameness	71 (24%)
Cutaneous lesions	178 (59.5%)	Conjunctivitis	30 (10%)
Weakness	171 (57.2%)	Dermatitis	31 (10%)
Urinary signs	81 (27.3%)	Ehrlichiosis	17 (5.7%)
Musculoskeletal signs	81 (27.1%)	CKD	74 (25%)
Gastrointestinal signs	80 (26.8%)	Ticks	35 (12%)
Ocular signs	61 (20.5%)	Gastroenteritis	55 (18%)
Pruritus	45 (15%)	Papilloma	15 (5.0%)
Pale mucous membranes	41 (14%)	Pyoderma	17 (5.7%)
Fever	22 (7.4%)	Uveitis	17 (5.7%)
Respiratory signs	20 (6.7%)	Xanthinuria	30 (10%)
Ear signs	15 (5.1%)		

CKD: chronic kidney disease

At admission, almost half of the dogs (120/284, 42.3%) showed medium positive IFAT titers (1/800–1/1600), while 29.2% (83/284) displayed high titers (> 1/1600). IFAT was low positive in 57/284 dogs (20.1%) and negative in 24 dogs (8.5%) (Table 3). Most dogs were classified as LeishVet stage III (116/299, 38.8%), followed by stage II (106/299, 35.5%), stage IV (41/299, 13.7%), and stage I (36/299, 12%) (Table 4).

**Table 4:** IFAT results and LeishVet clinical stage of the dogs included in the study

Characteristic	*n* = 300
IFAT	*n* (%)
Negative (< 1/200)	24 (8.5)
Low positive (1/200–1/400)	57 (20.1)
Medium positive (1/800–1/1600)	120 (42.3)
High positive (> 1/1600)	83 (29.2)
Unknown	16
LeishVet clinical stage	*n* (%)
I	36 (12.0)
II	106 (35.5)
III	116 (38.8)
IV	41 (13.7)
Unknown	1

### Specific therapy

In this study, we analyzed treatments prescribed for 300 dogs diagnosed with leishmaniosis between 2000 and 2022, focusing on three aspects: the first treatment administered after diagnosis, the overall treatment history, and treatment combinations used throughout the dogs’ lives (Table 5).

**Table 5:** Treatment protocols and medications used in dogs diagnosed with CanL

Characteristic	*n* = 300
Follow-up (years)	2.0 (2.0–4.0)
First treatment received	*n* (%)
MGA + ALLO	158 (52.7)
MIL + ALLO	37 (12.3)
ALLO	79 (26.3)
Prednisone	2 (0.7)
Others	24 (8.0)
Treatment history	
ALLO	291 (97.0)
MGA	210 (70.0)
MIL	70 (23.3)
Domperidone	51 (17)
Impromune^®^	12 (4.0)
Prednisone	50 (16.7)
Treatment combinations	
MGA + ALLO	208 (69.3)
MIL + ALLO	68 (22.7)

*MGA* meglumine antimoniate, *MIL* miltefosine, *ALLO* allopurinol

The table includes data on initial leishmanicidal treatment, full treatment history, and combination regimens. Follow-up is reported as median years (interquartile range, IQR)

Regarding the first treatment administered after diagnosis, most dogs received a combination of MGA plus ALLO (158/300, 52.7%), followed by ALLO monotherapy (79/300, 26.3%), and MIL plus ALLO (37/300, 12.3%) (Table 4). Only two dogs received prednisone alone as a first treatment after diagnosis.

Concerning overall treatment history, 97% of dogs were treated with ALLO at some point. Most dogs received MGA (210/300, 70%) as a leishmanicidal treatment, while 70 dogs (23.3%) were treated with MIL (Table 4). Among combination treatments, MGA plus ALLO was preferred in 69.3% of cases (208/300), followed by MIL plus ALLO (68/300, 22.7%) (Table 5). Other treatments included domperidone (51/300, 17%) and nucleotides (Impromune^®^) (12/300, 4%) as an immunomodulator. Prednisone alone was administered to 50/300 dogs (16.7%) at some point during the follow-up of their disease (Table 5).

### Outcome

The outcome was based on the number of leishmanicidal treatments received yearly due to a clinical relapse during the follow-up period or survival.

More than half of the dogs (154, 51.3%) had a good outcome, receiving less than 1 cycle of leishmanicidal treatment per year, while 66 dogs (22.0%) had a poor outcome, receiving one or more treatments per year (Table 6). Additionally, during the study period (2000–2022), 80 dogs (26.7%) died or were euthanized owing to CanL.

**Table 6:** Outcomes of dogs included in the study based on treatment frequency and survival

	*n* = 300
Outcome	*n* (%)
< 1 cycle per year	154 (51.3)
≥ 1 cycle per year	66 (22.0)
Died/euthanised	80 (26.7)

### Models

According to model 1 (Table 7), puppies (< 2 years) (odds ratio (OR) 0.07, confidence interval (CI) 0.01–0.48, *P* = 0.027) and mature adults (5–8 years) (OR 0.31, CI 0.12–0.73, *P* = 0.039) were less likely to require multiple leishmanicidal treatments than young adults (2–4 years). Dogs presenting weakness (OR 4.41, CI 1.38–15.9, *P* = 0.016) or those displaying ocular signs (OR 2.96, CI 1.03–8.60, *P* = 0.043) had higher odds of needing repeated treatment cycles. Conversely, dogs presenting weight loss had lower odds (OR 0.25, CI 0.06–0.85, *P* = 0.035).

**Table 7:** Logistic regression analysis of predictors of good (< 1 cycle per year) or poor (≥ 1 cycle per year) outcomes in CanL

	OR	95% CI	*P*-value
Age group			
Young adult	–	–	
Puppy	0.07	0.01–0.48	0.027^*^
Mature adult	0.31	0.12–0.73	0.039^*^
Senior	0.39	0.09–1.50	0.4
Collar			
No	–	–	
Yes	3.30	1.38–8.32	0.009^*^
Deworming			
No	–	–	
Yes	2.91	0.98–9.63	0.063^*^
Weight loss			
No	–	–	
Yes	0.25	0.06–0.85	0.035^*^
Weakness			
No	–	–	
Yes	4.41	1.38–15.9	0.016^*^
Musculoskeletal signs			
No	–	–	
Yes	0.40	0.12–1.17	0.11
Ocular signs			
No	–	–	
Yes	2.96	1.03–8.60	0.043^*^
Treated with domperidone			
No	–	–	
Yes	2.04	0.76–5.62	0.2
First cycle of treatment received			
MGA + ALLO	–	–	
ALLO	0.26	0.07–0.86	0.039^*^
MIL + ALLO	4.99	1.65–16.7	0.006^*^
Others	0.16	0.02–0.79	0.041^*^
Years			
2000–2010	–	–	
2011–2022	3.02	0.89–11.4	0.086

OR, odds ratio; CI, confidence interval. AIC = 184.07

*P < 0.05. *MGA* meglumine antimoniate, *MIL* miltefosine, *ALLO* allopurinol

Predictors of receiving ≥ 1 leishmanicidal treatment cycle per year are shown. Odds ratios (ORs), 95% confidence intervals (CIs), and *P*-values are reported

Furthermore, model 1 indicates that dogs first treated with MIL plus ALLO have five times higher odds of needing more treatment cycles than dogs receiving MGA plus ALLO (OR 4.99, CI 1.65–16.7, *P* = 0.006), while those receiving ALLO alone or other treatments have lower odds (OR 0.26, CI 0.07–0.86, *P* = 0.039). Finally, dogs treated between 2011 and 2022 had higher odds of receiving repeated treatment cycles than those treated between 2000 and 2010, even though this result was not statistically significant (OR 3.02, CI 0.89–11.04, *P* = 0.086).

Model 2 (Table 8), evaluating survival versus death/euthanasia, found that senior dogs have higher odds of death/euthanasia compared with young adults (OR 10.7, CI 3.80–32.5, *P* < 0.001), while medium (OR 4.63, CI 1.17–24.8, *P* = 0.046) and large-sized dogs (OR 4.45, 1.06–24.7, *P* = 0.060) have higher odds compared with small dogs. Puppies and mature adults showed no statistically significant difference in risk compared with young adults (Table 8). Regarding clinical signs, the odds of death/euthanasia due to CanL were higher in dogs with weakness (OR 2.92, CI 1.33–6.75, *P* = 0.009), gastrointestinal signs (OR 3.20, CI 1.48–7.14, *P* = 0.004), or lymphadenomegaly (OR 3.91, CI 1.68–9.79, *P* = 0.002).

**Table 8:** Logistic regression analysis of predictors of good (survival) or poor (death/euthanasia) outcomes in CanL

Characteristic	OR	95% CI	*P*-value
Age group			
Young adult	–	–	
Puppy	0.57	0.05–4.03	0.6
Mature adult	1.78	0.76–4.29	0.2
Senior	10.7	3.80–32.5	< 0.001^*^
Size			
Small	–	–	
Medium	4.63	1.17–24.8	0.046^*^
Large	4.45	1.06–24.7	0.060
Weakness			
No	–	–	
Yes	2.92	1.33–6.75	0.009^*^
Gastrointestinal signs			
No	–	–	
Yes	3.20	1.48–7.14	0.004^*^
Lymphadenomegaly			
No	–	–	
Yes	3.91	1.68–9.79	0.002^*^
LeishVet clinical Stage			
I	–	–	
II	0.55	0.14–2.80	0.4
III	0.81	0.21–4.07	0.8
IV	24.6	5.62–141	< 0.001^*^
Treated with domperidone			
No	–	–	
Yes	0.12	0.02–0.50	0.010^*^
Years			
2000–2010	–	–	
2011–2022	0.49	0.21–1.11	0.092

OR, odds Ratio; CI, confidence interval

*P < 0.05. AIC: 228.19

Odds ratios (ORs), 95% confidence intervals (CIs), and *P*-values are shown

**Fig. 2:**
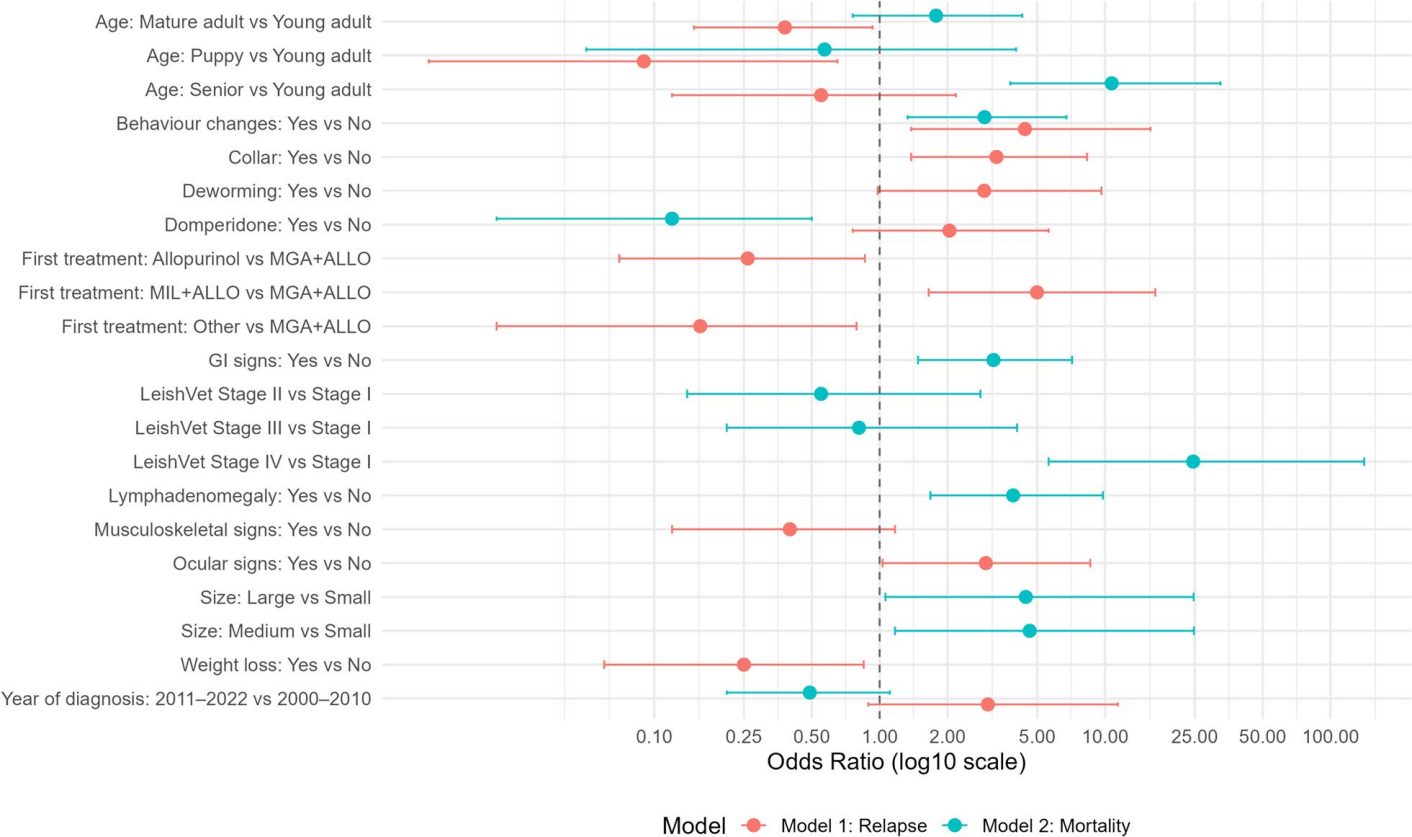
Forest plot of multivariable logistic regression analyses showing predictors of poor clinical outcome (≥ 1 treatment cycle/year; red) and mortality (death/euthanasia; blue) in dogs with CanL. Odds ratios and 95% confidence intervals are shown for each variable. Variables with ORs > 1 indicate increased odds of the outcome; ORs < indicate reduced odds. A dashed line at OR = 1 denotes no effect. A logarithmic scale to the *x*-axis was applied to improve the visualization of effect sizes. OR, CI, and *P*-values of each variable are displayed in Tables 7 and 8

Furthermore, dogs in LeishVet stage IV had 24.6 times higher odds of death/euthanasia compared with those in stage I (OR 24.6, 5.62–141, *P* < 0.001). Stages II and III did not show a significant difference from stage I. Interestingly, dogs treated between 2011 and 2022 had lower odds of death/euthanasia than those treated between 2000 and 2010, although this result was not statistically significant (OR 0.49, CI 0.21–1.11, *P* = 0.092). Finally, our results indicate that dogs treated with domperidone have lower odds of death/euthanasia due to CanL (OR 0.12, CI 0.02–0.50, *P* = 0.010). A forest plot summarizing the adjusted odds ratios and 95% confidence intervals is presented in Fig. 2.

## Discussion

This 20-year retrospective study identified key risk factors for poor prognosis and mortality in CanL by integrating demographic, clinical, clinicopathological, and therapeutic variables into predictive models. Given the complex and variable clinical course of the disease, ranging from long-term remission to frequent relapses or early death, our findings provide evidence to support clinical decision-making and potentially improve patient outcomes.

Among demographic variables, mature adults (4–8 years) were about 62% less likely to receive multiple treatment cycles than young adult dogs. The association of age with infection or disease in CanL has been investigated in several studies, yielding conflicting results [41–46]. However, two large epidemiological studies noted a bimodal distribution of sick animals, with a peak around 3 years and a less pronounced one around 8 years [41, 47]. Additionally, recent research found that young adult dogs display higher circulating immune complex levels [48], which are associated with more severe cases of CanL [13, 49]. Other factors may also contribute to the increased risk in young adult dogs. They typically spend more time outdoors than puppies and seniors, increasing their exposure to vectors. Furthermore, while their immune systems are more developed than those of puppies, they may not be as robust or well-regulated as mature dogs.

Medium- to large-sized dogs showed a fourfold higher mortality risk than small dogs. Even though no prior studies have investigated the association between body size and mortality in CanL, it was found that larger dogs are at a higher risk of infection [47, 50] and of developing severe forms of the disease [31]. The increased mortality risk found in our study can be attributed to a greater time spent outdoors, leading to more exposure to vector bites, and to pet owners potentially being less aware of clinical signs, delaying diagnosis and treatment [51, 52].

Regarding epidemiological variables, using repellents or regular deworming was associated with an increased risk of requiring repeated treatment cycles. However, this association may reflect more frequent veterinary visits and consistent prescriptions for preventive measures.

Interestingly, dogs treated in 2011–2022 had higher odds of receiving repeated treatment cycles but lower odds of death or euthanasia due to CanL than those treated in 2000–2010, even though these differences were not statistically significant. This trend might be attributed to advancements in CanL management, increased awareness, and improved supportive care.

Historically, MGA plus ALLO were the mainstays of therapy in CanL. Over the last two decades, MIL has been introduced and is now widely used [2, 15, 53, 54], including in Brazil since 2017, where euthanasia of infected dogs was used as a disease control strategy despite limited scientific evidence supporting its effectiveness. More recently, there has been a shift toward a multimodal approach, including the use of immunotherapy and preventive strategies such as immunomodulators, vaccines, and ectoparasiticides, reflecting a broader “One Health” perspective [55–57]. International guidelines developed and periodically updated by the LeishVet group have contributed to more unified and evidence-based treatment protocols, emphasizing the importance of individualized therapy according to clinical staging and comorbidities. Despite this, in light of our results, the potential for drug resistance over the last decade should also be considered [27, 58, 59].

Quantitative serology is one of the most useful techniques for diagnosing CanL [60]. Previous studies reported an association between high antibody titers and clinical disease [16, 61, 62], as well as an increased risk of disease progression to more advanced stages [31]. In the current study, antibody levels were not associated with an increased risk of multiple leishmanicidal treatment cycles or mortality, suggesting that they are not a good biomarker of prognosis in CanL when other, more significant variables are considered. This aligns with a recent study that found that antibody levels were not a good predictor of relapses in CanL in the presence of a low albumin/globulins ratio and clinical signs [17]. Moreover, a study evaluating dogs with CanL in a nonendemic country did not find a correlation between antibody levels and survival time [63]. Thus, antibody titers should always be interpreted along with other clinicopathological variables to assess treatment response and prognosis.

In CanL, the appearance of clinical signs is associated with an ineffective cellular immune response, and parasite multiplication and dissemination [64]. Here, dogs displaying weakness and those with ocular signs were at higher risk of requiring multiple treatment cycles. Surprisingly, dogs showing weight loss were at lower risk. This unexpected finding might be attributed to increased pet owners’ awareness and earlier diagnosis of more noticeable signs, which could lead to prompt intervention during clinical relapse. Moreover, weight loss is one of the most common clinical signs in CanL and is usually present in earlier stages of the disease [65]. Conversely, ocular signs, which affect up to two-thirds of sick dogs [66, 67], typically appear later as the disease progresses and are associated with immune complex formation and a worse prognosis [13, 48, 65].

Regarding mortality, dogs showing weakness, gastrointestinal signs, or lymphadenomegaly had a threefold higher risk of death than dogs not displaying these clinical signs. Prior research suggests that dogs with systemic signs have a predominant nonprotective humoral response, associated with increased parasite burden and dissemination to multiple organs [48, 49, 64]. Specifically, lymphadenomegaly has been associated with increased parasite loads and more severe clinical manifestations in CanL [20, 68]. Notably, a case–control study from Brazil found that diarrhea was associated with an eightfold increase in mortality risk [69]. In CanL, gastrointestinal signs are present in up to 30% of dogs and can be secondary to kidney or liver disease [70]. Furthermore, dogs with gastrointestinal signs may have a poorer tolerance to therapeutic agents, including leishmanicidal drugs, which could contribute to their increased mortality risk.

A clinical staging system based on the severity of clinicopathological abnormalities and antibody levels has been proposed by the LeishVet group to guide practitioners in the management of CanL [13]. Even though in our study only 13% of dogs were classified as LeishVet stage IV (very severe disease, characterized by the presence of end-stage renal disease), these dogs had 24.6 times higher odds of death/euthanasia than those in stage I. Stages II and III did not show a significant difference from stage I. This result confirms that end-stage kidney disease is the main cause of death in dogs with CanL [71, 72], reinforcing the importance of early and continuous monitoring of renal function in these patients.

In Europe, MGA plus ALLO is considered the first-line treatment for CanL [18]. In our study, over half of the dogs were first treated with MGA plus ALLO, while 12.3% received MIL plus ALLO. Remarkably, receiving MIL plus ALLO as a first-line treatment was associated with a fivefold higher risk of requiring multiple leishmanicidal cycles. Although studies comparing these treatments are scarce, one long-term study found that dogs treated with MGA plus ALLO showed more stable clinicopathological findings and fewer relapses than those treated with MIL plus ALLO [61]. In contrast, previous comparative studies have demonstrated that both combination protocols yield significant clinical improvements and reductions in parasite load, with no statistically significant differences in efficacy over short- to medium-term follow-up [2, 53]. Moreover, poorer outcomes in dogs treated with MIL + ALLO could also be the result of baseline disease severity, as this treatment combination is often used in dogs with renal disease. Nevertheless, the results of the current study, which includes a large sample size, long follow-up periods, and logistic regression analysis, suggest that choosing MGA plus ALLO as a first-line treatment may offer more durable long-term disease control than MIL plus ALLO. Further randomized, blinded, multicentric studies are needed to confirm our findings.

Current guidelines published by the LeishVet group recommend allopurinol (ALLO) as maintenance therapy for 6–12 months after an initial 28-day course of combination treatment with leishmanicidal drugs (MGA or MIL) [13]. However, ALLO is sometimes used as a primary treatment in specific scenarios, such as in dogs with mild clinical signs, advanced renal disease, intolerance to leishmanicidal drugs, or in countries where such drugs are unavailable [18]. In our study, dogs that received ALLO monotherapy as a first-line treatment had 74% lower odds of requiring repeated leishmanicidal treatments than those treated with MGA + ALLO. Similarly, a recent retrospective study by Jong et al. [73] reported that 3 months of ALLO monotherapy was effective in more than half of the CanL cases evaluated. Despite these findings, caution is warranted when interpreting results from retrospective studies, as they are not sufficient to support a broad recommendation for ALLO monotherapy as an initial treatment for CanL. Moreover, the development of resistance to ALLO has been documented [28, 74–76], and prior studies have shown that ALLO alone is less effective than combination treatments and often insufficient to achieve parasite clearance [77–81]. Therefore, combination therapies remain the most effective and evidence-based approach for treating dogs with CanL [2, 18, 82–84].

In this study, only two dogs referred to our service had been treated with prednisone alone immediately after diagnosis. This result is reassuring, as prednisone is not recommended as a first-line treatment for CanL, particularly when used as a standalone therapy. While corticosteroids may be necessary to manage severe clinical signs linked to immune complex deposition, such as epistaxis and uveitis [85], it is advisable to avoid immunosuppressive doses and prolonged use owing to the high risk of adverse effects. From our experience, a dosing regimen of 0.5 mg/kg once daily (q24h) for 1–2 weeks, followed by a gradual reduction over a maximum of two additional weeks, is both effective and safe in most cases. Dogs that do not respond to this protocol should be referred to a specialist in infectious diseases before receiving more aggressive treatment options. We emphasize the urgent need for clear guidelines on the responsible use of corticosteroids in CanL.

Domperidone, a dopamine D2 receptor antagonist, has been shown to enhance the innate-cell-mediated immune response, which is crucial for controlling *Leishmania* infection and preventing the progression to clinical disease. A controlled, randomized clinical trial found that dogs treated with domperidone had a sevenfold lower risk of developing clinical disease than untreated dogs [3]. Additionally, an uncontrolled study reported reductions in IFAT titers, globulins, and gamma globulins in dogs treated with domperidone [5]. More recently, a blinded, randomized, controlled, multicenter clinical trial demonstrated that low-seropositive dogs treated with domperidone were less likely to develop clinical disease and presented lower parasitemia than the placebo group [86]. In our study, dogs that received domperidone had 0.12 lower odds of death or euthanasia due to CanL, translating to an 88% reduction in risk. Although most dogs were in LeishVet stage I, our findings suggest that domperidone may reduce mortality risk in CanL, likely by preventing or slowing disease progression. However, the role of domperidone as both a prophylactic and therapeutic agent remains ambiguous, and the optimal timing for its administration is unclear. Moreover, its impact on the infectivity of dogs to sandfly vectors has not been evaluated in previous studies [87], which is particularly relevant given that the only randomized controlled trial found no significant differences in parasite load between treated and placebo groups [86].

Impromune^®^, an active hexose-correlated compound (AHCC) derived from the mycelia of shiitake mushrooms (*Lentinula edodes*), has been shown to enhance Th1 responses and modulate the activity of intestinal epithelial cells and macrophages, likely through the regulation of Toll-like receptor signaling pathways [88]. Two randomized controlled trials demonstrated that this compound has good efficacy for the treatment of CanL, preventing disease progression [6, 7]. In our study, only 4% of the dogs received Impromune^®^, limiting our ability to assess its impact on prognosis. However, given its proven immunomodulatory properties, clinicians should consider its use, particularly in dogs with xanthinuria [69].

It is important to acknowledge the limitations of our study. As in all retrospective studies, data recording and cleaning may affect the quality of our findings. Also, owing to time and cost constraints, the raw data of laboratory parameters and antiproteinuric treatment were not recorded. Finally, since the study population was limited to referred cases of CanL at our veterinary teaching referral hospital, the model’s performance may vary in other populations. Despite these limitations, our study identifies relevant risk factors for poor outcomes in a large cohort over 20 years.

## Conclusions

Our results suggest that young adults, larger dogs, and those displaying weakness or ocular signs are at increased risk of receiving multiple leishmanicidal treatments due to clinical relapse. Additionally, this risk was higher in dogs treated with miltefosine plus allopurinol as a first-line treatment. Mortality risk was higher in dogs in LeishVet stage IV and those displaying weakness, gastrointestinal signs and lymphadenomegaly, while treatment with domperidone appears to reduce this risk. These insights enhance our understanding of the demographic, epidemiological, and clinical factors associated with prognosis in CanL and provide valuable guidance for improved clinical management and treatment-related decision-making.

## Supplementary Information

Supplementary material 1

Supplementary material 2

## Data Availability

Data supporting the main conclusions of this study are included in the manuscript.

## Abbreviations

CanL: Canine leishmaniosis
MGA: Meglumine antimoniate
MIL: Miltefosine
ALLO: Allopurinol
IFAT: Indirect immunofluorescence antibody test
PCR: Polymerase chain reaction
AIC: Akaike information criterion
VIF: Variance inflation factor
