# Supplementary material for: Unveiling prognostic indicators in canine leishmaniosis: two decades of evidence
Source: Parasit Vectors. 2025 Nov 18;18:467. doi: 10.1186/s13071-025-07042-0 (PMC12625734; doi:10.1186/s13071-025-07042-0)
Supplement: Supplementary file 1 — Supplementary material 1 [file 13071_2025_7042_MOESM1_ESM.docx]

**Supplementary Table 1.** Univariate logistic regression analysis of demographic, clinical, and therapeutic variables evaluated as predictors of relapse (≥1 leishmanicidal treatment cycle per year) in dogs with CanL. Variables with a *P*-value < 0.25 were considered eligible for inclusion in the multivariate logistic regression as described by Bursac et al. (2008) [37]. Odds ratios (OR), 95% confidence intervals, and *P*-values are shown for each variable. Eighty dogs that died or were euthanized due to CanL were excluded.

| **Variables** | **N** | **OR**^1^ | **95% CI**^1^ | ***P*-value** |
| --- | --- | --- | --- | --- |
| Sex | 220 |  |  |  |
| Female |  | — | — |  |
| Male |  | 0.88 | 0.48-1.63 | 0.69 |
| Age | 220 |  |  |  |
| Mature adult |  | — | — |  |
| Puppy |  | 0.55 | 0.08-2.20 | 0.45 |
| Senior |  | 0.86 | 0.26-2.44 | 0.79 |
| Young adult |  | 1.61 | 0.84-3.11 | 0.15 |
| Breed | 220 |  |  |  |
| Crossbreed |  | — | — |  |
| Purebred |  | 0.91 | 0.48-1.77 | 0.77 |
| Habitat | 218 |  |  |  |
| Indoors |  | — | — |  |
| Outdoors |  | 0.65 | 0.32-1.25 | 0.21 |
| Size | 219 |  |  |  |
| Small |  | — | — |  |
| Medium |  | 0.84 | 0.36-2.04 | 0.69 |
| Large |  | 1.19 | 0.51-2.94 | 0.69 |
| Weight loss | 220 |  |  |  |
| No |  | — | — |  |
| Yes |  | 1.93 | 1.03-3.75 | 0.046 |
| Fever | 220 |  |  |  |
| No |  | — | — |  |
| Yes |  | 0.68 | 0.15-2.24 | 0.56 |
| Weakness | 220 |  |  |  |
| No |  | — | — |  |
| Yes |  | 2.39 | 1.28-4.60 | 0.007 |
| Gastrointestinal signs | 220 |  |  |  |
| No |  | — | — |  |
| Yes |  | 0.73 | 0.32-1.54 | 0.42 |
| Respiratory signs | 220 |  |  |  |
| No |  | — | — |  |
| Yes |  | 1.13 | 0.30-3.52 | 0.85 |
| Urinary signs | 219 |  |  |  |
| No |  | — | — |  |
| Yes |  | 1.60 | 0.78-3.18 | 0.19 |
| Cutaneous signs | 220 |  |  |  |
| No |  | — | — |  |
| Yes |  | 1.37 | 0.75-2.58 | 0.31 |
| Pruritus | 220 |  |  |  |
| No |  | — | — |  |
| Yes |  | 1.49 | 0.65-3.25 | 0.33 |
| Ectoparasites | 220 |  |  |  |
| No |  | — | — |  |
| Yes |  | 3.12 | 1.16-8.43 | 0.023 |
| Pale mucous membranes | 218 |  |  |  |
| No |  | — | — |  |
| Yes |  | 0.46 | 0.10-1.43 | 0.23 |
| Lymphadenomegaly | 219 |  |  |  |
| No |  | — | — |  |
| Yes |  | 2.53 | 1.34-4.97 | 0.005 |
| Ear signs | 219 |  |  |  |
| No |  | — | — |  |
| Yes |  | 0.92 | 0.20-3.22 | 0.90 |
| Musculoskeletal signs | 220 |  |  |  |
| No |  | — | — |  |
| Yes |  | 0.51 | 0.23-1.06 | 0.083 |
| Ocular signs | 219 |  |  |  |
| No |  | — | — |  |
| Yes |  | 1.82 | 0.85-3.78 | 0.11 |
| Arthrosis | 220 |  |  |  |
| No |  | — | — |  |
| Yes |  | 1.32 | 0.62-2.73 | 0.45 |
| Lameness | 220 |  |  |  |
| No |  | — | — |  |
| Yes |  | 0.98 | 0.46-1.96 | 0.95 |
| Conjunctivitis | 220 |  |  |  |
| No |  | — | — |  |
| Yes |  | 0.68 | 0.19-1.94 | 0.50 |
| Dermatitis | 220 |  |  |  |
| No |  | — | — |  |
| Yes |  | 1.36 | 0.53-3.27 | 0.50 |
| Ehrlichiosis | 220 |  |  |  |
| No |  | — | — |  |
| Yes |  | 0.75 | 0.16-2.50 | 0.67 |
| Chronic Kidney Disease | 220 |  |  |  |
| No |  | — | — |  |
| Yes |  | 1.02 | 0.47-2.10 | 0.96 |
| Ticks | 220 |  |  |  |
| No |  | — | — |  |
| Yes |  | 0.92 | 0.35-2.21 | 0.86 |
| Gastroenteritis | 220 |  |  |  |
| No |  | — | — |  |
| Yes |  | 1.74 | 0.82-3.61 | 0.14 |
| Pyoderma | 220 |  |  |  |
| No |  | — | — |  |
| Yes |  | 0.00 |  | 0.98 |
| Uveitis | 220 |  |  |  |
| No |  | — | — |  |
| Yes |  | 3.57 | 1.14-11.6 | 0.028 |
| Xanthine | 220 |  |  |  |
| No |  | — | — |  |
| Yes |  | 1.20 | 0.47-2.84 | 0.68 |
| Pipette | 215 |  |  |  |
| No |  | — | — |  |
| Yes |  | 2.27 | 1.22-4.27 | 0.010 |
| Collar | 215 |  |  |  |
| No |  | — | — |  |
| Yes |  | 3.75 | 1.97-7.45 | <0.001 |
| Deworming | 216 |  |  |  |
| No |  | — | — |  |
| Yes |  | 5.20 | 2.35-13.2 | <0.001 |
| LeishVet Stage | 220 |  |  |  |
| I |  | — | — |  |
| II |  | 1.59 | 0.61-4.70 | 0.36 |
| III |  | 1.83 | 0.71-5.35 | 0.23 |
| IV |  | 2.25 | 0.39-11.5 | 0.33 |
| Treated with domperidone | 220 |  |  |  |
| No |  | — | — |  |
| Yes |  | 2.41 | 1.22-4.73 | 0.010 |
| First treated with ALLO+MGA^2^ | 220 |  |  |  |
| No |  | — | — |  |
| Yes |  | 1.03 | 0.57-1.90 | 0.91 |
| First treated with ALLO+MIL^2^ | 220 |  |  |  |
| No |  | — | — |  |
| Yes |  | 6.69 | 2.99-15.7 | <0.001 |
| First treatment | 220 |  |  |  |
| ALLO |  | — | — |  |
| ALLO+MGA |  | 4.56 | 1.68-16.0 | 0.007 |
| ALLO+MIL |  | 21.2 | 6.53-85.1 | <0.001 |
| Others |  | 2.30 | 0.42-11.5 | 0.31 |
| IFAT | 216 |  |  |  |
| High positive |  | — | — |  |
| Low positive |  | 0.56 | 0.21-1.41 | 0.23 |
| Medium positive |  | 1.07 | 0.53-2.18 | 0.86 |
| Negative |  | 0.28 | 0.04-1.12 | 0.11 |
| Visit range | 220 |  |  |  |
| 2000-2010 |  | — | — |  |
| 2011-2022 |  | 5.01 | 2.27-12.7 | <0.001 |
| ^1^OR = Odds Ratio, CI = Confidence Interval. ^2^ALLO: allopurinol, MGA: meglumine antimoniate, MIL: miltefosine. | | | | |
