# Supplementary material for: Unveiling prognostic indicators in canine leishmaniosis: two decades of evidence
Source: Parasit Vectors. 2025 Nov 18;18:467. doi: 10.1186/s13071-025-07042-0 (PMC12625734; doi:10.1186/s13071-025-07042-0)
Supplement: Supplementary file 2 — Supplementary material 2 [file 13071_2025_7042_MOESM2_ESM.docx]

**Supplementary Table 2.** Univariate logistic regression analysis of demographic, clinical, and therapeutic variables evaluated as predictors of mortality (death or euthanasia) in dogs with CanL. Variables with a *P*-value < 0.25 were considered eligible for inclusion in the multivariate logistic regression model as described by Bursac et al. (2008) [37]. Odds ratios, 95% confidence intervals, and *P*-values are shown for each variable.

| **Characteristic** | **N** | **OR**^1^ | **95% CI**^1^ | ***P*-value** |
| --- | --- | --- | --- | --- |
| Sex | 300 |  |  |  |
| Female |  | — | — |  |
| Male |  | 1.29 | 0.76-2.21 | 0.35 |
| Age | 300 |  |  |  |
| Mature adult |  | — | — |  |
| Puppy |  | 0.41 | 0.06-1.56 | 0.25 |
| Senior |  | 2.85 | 1.43-5.72 | 0.003 |
| Young adult |  | 0.68 | 0.36-1.26 | 0.22 |
| Dog breed | 300 |  |  |  |
| Crossbreed |  | — | — |  |
| Purebred |  | 1.82 | 0.99-3.52 | 0.064 |
| Habitat | 292 |  |  |  |
| Indoors |  | — | — |  |
| Outdoors |  | 1.33 | 0.77-2.30 | 0.30 |
| Size | 299 |  |  |  |
| Small |  | — | — |  |
| Medium |  | 2.78 | 1.10-8.52 | 0.046 |
| Large |  | 3.65 | 1.43-11.3 | 0.012 |
| Weight loss | 299 |  |  |  |
| No |  | — | — |  |
| Yes |  | 1.56 | 0.91-2.73 | 0.11 |
| Fever | 299 |  |  |  |
| No |  | — | — |  |
| Yes |  | 1.33 | 0.49-3.29 | 0.55 |
| Weakness | 299 |  |  |  |
| No |  | — | — |  |
| Yes |  | 1.76 | 1.04-3.05 | 0.039 |
| Gastrointestinal signs | 299 |  |  |  |
| No |  | — | — |  |
| Yes |  | 2.86 | 1.65-4.97 | <0.001 |
| Respiratory signs | 299 |  |  |  |
| No |  | — | — |  |
| Yes |  | 1.21 | 0.42-3.14 | 0.71 |
| Urinary signs | 297 |  |  |  |
| No |  | — | — |  |
| Yes |  | 2.83 | 1.63-4.92 | <0.001 |
| Cutaneous signs | 299 |  |  |  |
| No |  | — | — |  |
| Yes |  | 1.24 | 0.73-2.12 | 0.43 |
| Pruritus | 299 |  |  |  |
| No |  | — | — |  |
| Yes |  | 1.01 | 0.48-2.03 | 0.97 |
| Ectoparasites | 300 |  |  |  |
| No |  | — | — |  |
| Yes |  | 1.42 | 0.59-3.24 | 0.41 |
| Pale mucous membranes | 297 |  |  |  |
| No |  | — | — |  |
| Yes |  | 3.58 | 1.82-7.11 | <0.001 |
| Lymphadenomegaly | 295 |  |  |  |
| No |  | — | — |  |
| Yes |  | 1.77 | 1.02-3.13 | 0.046 |
| Ear signs | 297 |  |  |  |
| No |  | — | — |  |
| Yes |  | 0.69 | 0.15-2.24 | 0.57 |
| Musculoskeletal signs | 299 |  |  |  |
| No |  | — | — |  |
| Yes |  | 1.25 | 0.70-2.18 | 0.44 |
| Ocular signs | 297 |  |  |  |
| No |  | — | — |  |
| Yes |  | 1.99 | 1.08-3.61 | 0.024 |
| Arthrosis | 300 |  |  |  |
| No |  | — | — |  |
| Yes |  | 1.06 | 0.54-1.98 | 0.86 |
| Lameness | 300 |  |  |  |
| No |  | — | — |  |
| Yes |  | 1.21 | 0.66-2.16 | 0.53 |
| Conjunctivitis | 300 |  |  |  |
| No |  | — | — |  |
| Yes |  | 1.43 | 0.61-3.14 | 0.39 |
| Dermatitis | 300 |  |  |  |
| No |  | — | — |  |
| Yes |  | 0.63 | 0.23-1.51 | 0.33 |
| Ehrlichiosis | 300 |  |  |  |
| No |  | — | — |  |
| Yes |  | 0.57 | 0.13-1.82 | 0.39 |
| Chronic kidney disease | 300 |  |  |  |
| No |  | — | — |  |
| Yes |  | 2.21 | 1.26-3.87 | 0.006 |
| Ticks | 300 |  |  |  |
| No |  | — | — |  |
| Yes |  | 0.66 | 0.26-1.49 | 0.35 |
| Gastroenteritis | 300 |  |  |  |
| No |  | — | — |  |
| Yes |  | 1.16 | 0.59-2.19 | 0.65 |
| Papilloma | 300 |  |  |  |
| No |  | — | — |  |
| Yes |  | 1.40 | 0.42-4.08 | 0.55 |
| Pyoderma | 300 |  |  |  |
| No |  | — | — |  |
| Yes |  | 1.54 | 0.52-4.20 | 0.41 |
| Uveitis | 300 |  |  |  |
| No |  | — | — |  |
| Yes |  | 0.84 | 0.23-2.45 | 0.76 |
| Xanthine | 300 |  |  |  |
| No |  | — | — |  |
| Yes |  | 0.28 | 0.07-0.82 | 0.040 |
| Pipette | 291 |  |  |  |
| No |  | — | — |  |
| Yes |  | 0.74 | 0.42-1.26 | 0.27 |
| Collar | 290 |  |  |  |
| No |  | — | — |  |
| Yes |  | 0.92 | 0.54-1.56 | 0.75 |
| Deworming | 289 |  |  |  |
| No |  | — | — |  |
| Yes |  | 1.06 | 0.61-1.89 | 0.83 |
| LeishVet Stage | 299 |  |  |  |
| I |  | — | — |  |
| II |  | 2.25 | 0.70-10.0 | 0.22 |
| III |  | 3.18 | 1.03-13.9 | 0.072 |
| IV |  | 39.1 | 11.0-192 | <0.001 |
| Treated with domperidone | 300 |  |  |  |
| No |  | — | — |  |
| Yes |  | 0.09 | 0.01-0.30 | 0.001 |
| First treated with ALLO+MGA | 300 |  |  |  |
| No |  | — | — |  |
| Yes |  | 0.75 | 0.45-1.26 | 0.28 |
| First treated with ALLO+MIL | 300 |  |  |  |
| No |  | — | — |  |
| Yes |  | 0.61 | 0.24-1.37 | 0.26 |
| First treatment | 300 |  |  |  |
| ALLO |  | — | — |  |
| ALLO+MGA |  | 0.66 | 0.36-1.20 | 0.17 |
| ALLO+MIL |  | 0.48 | 0.17-1.18 | 0.12 |
| Others |  | 0.97 | 0.37-2.39 | 0.94 |
| IFAT | 284 |  |  |  |
| High positive |  | — | — |  |
| Low positive |  | 0.99 | 0.45-2.12 | 0.98 |
| Medium positive |  | 0.77 | 0.40-1.48 | 0.43 |
| Negative |  | 0.73 | 0.22-2.07 | 0.57 |
| Visit range | 300 |  |  |  |
| 2000-2010 |  | — | — |  |
| 2011-2022 |  | 0.47 | 0.28-0.79 | 0.005 |
| ^1^OR = Odds Ratio, CI = Confidence Interval. ^2^ALLO: allopurinol, MGA: meglumine antimoniate, MIL: miltefosine. | | | | |
